# Socially Shared Feelings of Imminent Recall: More Tip-of-the-Tongue States Are Experienced in Small Groups

**DOI:** 10.3389/fpsyg.2021.704433

**Published:** 2021-07-16

**Authors:** Luc Rousseau, Nathalie Kashur

**Affiliations:** Department of Psychology, Laurentian University, Greater Sudbury, ON, Canada

**Keywords:** social cognition, metacognition, tip-of-the-tongue states, group recall, social contagion effect, collective memory

## Abstract

Tip-of-the-tongue (TOT) states are typically defined as feelings of imminent recall for known, but temporarily inaccessible target words. However, TOTs are not merely instances of retrieval failures. Clues that increase the subjective likelihood of retrieval success, such as cue familiarity and target-related information, also have been shown to elicit feelings of imminent recall, supporting a metacognitive, inferential etiology of the TOT phenomenon. A survey conducted on our university campus provided anecdotal evidence that TOTs are occasionally shared among people in small groups. Although shared TOTs may suggest the influence of social contagion, we hypothesized that metacognitive appraisal of group recall efficiency could be involved. There should be more instances of remembering in several heads than in one. From this, we conjectured that people remembering together entertain the inference that successful retrieval is more likely in group recall than in a single-person recall situation. Such a metacognitive appraisal may drive a stronger feeling of closeness with the target word and of recall imminence, precipitating one (or more people) into a TOT state. We used general knowledge questions to elicit TOTs. We found that participants reported more TOTs when remembering in small groups than participants remembering alone. Critically, the experimental manipulation selectively increased TOTs without affecting correct recall, suggesting that additional TOTs observed in small groups were triggered independently from the retrieval process. Near one third (31%) of the TOTs in small groups were reported by two or more participants for the same items. However, removing common TOTs from the analyses did not change the basic pattern of results, suggesting that social contagion was not the main factor involved in the observed effect. We argue that beyond social contagion, group recall magnifies the inference that target words will be successfully retrieved, prompting the metacognitive monitoring system to launch more near-retrieval success “warning” (TOT) signals than in a single-person recall situation.

## Introduction

Remembering is a social as well as an individual activity. In the past three decades, a dynamic new field has emerged, referred to as *socially shared cognition* (Resnick et al., 1991) or *socially distributed remembering* (Sutton et al., 2010). As private as they may seem, memory processes are not immune to social influences, as demonstrated by the *memory conformity*, or “social contagion” of memory effect (Roediger et al., 2001), the *collaborative inhibition effect* (Weldon and Bellinger, 1997), as well as the *socially shared retrieval-induced forgetting effect* (Cuc et al., 2007). Surprisingly, despite its ubiquity in memory research, the tip-of-the-tongue (henceforth TOT) phenomenon remains largely unexplored outside the traditional, single-person recall paradigm introduced 55 years ago by Brown and McNeill (1966).

Schwartz and Cleary (2016) rightfully stated that understanding the social dynamics of TOTs is yet to be addressed. As they pointed out, in the real world, TOTs often occur in social situations and people discussing a shared memory (e.g., a movie seen together) could simultaneously experience a TOT (e.g., for the name of the main actress). When one is struggling with a TOT, nearby people are often the first to be called upon for help (before turning to Google). Yet, it is not uncommon to realize that the unfortunate bystanders fall into the same “TOT trap”! To get a rough idea of the naturalistic prevalence of shared TOTs within small groups, we have conducted a small survey on the Laurentian University campus in Sudbury, Ontario, Canada. A total of 197 participants, mostly undergraduate students, were submitted two written questions:

1. *While trying to remember something, did you ever experience having a word “on the tip of your tongue”? You were quite sure to know the word (for example: an actor's name) and it was on the verge of coming back to you, but you were unable to recall it for some time*.
2. *Over the past 6 months, are you aware of an occasion when someone, in a small group, had a word on the tip of their tongue, and, all of a sudden, others in the group also happened to have the same word on the tip of their tongue? For some time, nobody in the group could recall the word. Maybe you were the first one with the word on the tip of your tongue, or maybe you “caught” it from another person*.

Expectedly, 97.5% of the polled population answered *yes* to the first question. Of those respondents, 95.5% also answered *yes* to the second question, 11.7% indicating one *occasion*, and 83.8% indicating *several occasions*. Thus, anecdotal evidence suggests that shared TOTs do occasionally occur in small groups.

### Social Influences on TOTs

That TOT prevalence could be enhanced by social factors has been suggested before. In his landmark review of the first 25 years of research on the TOT phenomenon, Brown (1991) found himself puzzled by the fact that, regardless of the target word's type and difficulty level, TOT rates were remarkably consistent across published studies. He reasoned that such a consistency “may reflect subjects' acquiescence to the experimenter's suggestion that TOTs will be occasionally experienced, and such demand characteristics may be more pronounced when subjects are tested in groups and witness others experiencing TOTs” (p. 208). Incidentally, and seldom noticed, in Brown and McNeill's (1966) seminal study, participants were tested in groups (56 participants attended one of three group testing sessions). Although participants filled out their own response sheet, they were also instructed to raise their hand when experiencing a TOT. Therefore, several participants witnessed others experiencing TOTs. However, the extent to which such a procedure is likely to induce “more pronounced” demand characteristics is still currently unknown.

In turn, Widner et al. (1996) provided compelling evidence that experimenter-induced demand characteristics play a role in the TOT phenomenon. Two groups of participants received an identical set of general knowledge questions. However, instructions differed slightly between the groups. In the *high demand characteristic* (HD) condition, participants were instructed that the questions were normatively easy to answer, while in the *low demand characteristics* (LD) condition, participants were instructed that the questions were normatively difficult to answer. It was hypothesized that participants in the HD condition would feel more pressured to answer questions than those in the LD condition because they would fear to appear less knowledgeable by failing to answer normatively easy questions. There was a remarkable, 200% TOT increase from the LD (3.6%) to the HD (10.8%) conditions. Target retrievability could not account for this finding, because correct recall did not differ between the HD and the LD conditions. Widner et al. (1996) interpreted their results as reflecting the adoption, by participants in the HD condition, of a more liberal criterion to report TOTs. The authors raised a second, alternative, interpretation: Anxiety induced by the experimenter's social pressure to answer normatively easy questions may have induced more TOTs. This social stress account of Widner et al. (1996) data was also advocated by Schwartz (2002), who compared it to the embarrassing situation in which one inadvertently encounters a familiar person without being able to remember their name.

A direct investigation of the effects of social stress on TOTs was conducted by James et al. (2018). In the *Trier Social Stress Test* (TSST) condition, participants were told that a psychologist with an expertise in non-verbal behavior would be analyzing their body language through a one-way mirror. A recording of the expert's voice was played over to add credibility to the situation. The experimenter, present in the room, asked the participant to look directly at the one-way mirror while setting up video and audio recording devices. Participants performed three consecutive tasks under third-party observation: delivering a 5-min speech for a mock job interview without access to notes, doing mental subtractions aloud for 5 min with the experimenter giving live feedback on all errors, and providing answers to 60 rare word definitions. In the placebo condition (pTSST), participants performed less stressful preliminary tasks (a speech on their favorite vacation spot, with access to notes, and a subtraction task on paper, without feedback), but rare word definitions were identical. Critically, in the placebo condition, there was no mention of a third-party, expert observer, blinds covered the one-way mirror, and the video and audio recording devices were removed from the room. TOTs increased from 4 to 6% under social stress. James et al. (2018) interpreted this finding as reflecting a stress-induced deficit in the transmission of activation from semantic to phonological nodes (Burke et al., 1991). If this was the case, though, the experimental manipulation would also have hindered retrievability. But correct recall did not differ between the TSST and the pTSST conditions. This pattern of results, later replicated in a sample of elderly participants (Schmank and James, 2020), suggests, rather, that social stress selectively increased TOTs independently from the retrieval process.

Although previous studies provided evidence that social factors do influence TOTs (Widner et al., 1996; James et al., 2018; Schmank and James, 2020), they were still based on a single-person recall setting. No published study has yet addressed factors underlying the co-occurrence of TOTs in small groups.

### Two Distinct Factors Than Can Potentially Induce TOTs in Small Groups: Social Contagion and Metacognitive Appraisal of Group Recall Efficiency

A peculiar feeling may arise when one is experiencing a TOT next to someone else experiencing a TOT. To get back to the social situation described in our survey, it is the feeling of having “caught” the TOT, as if TOT states were contagious. Schwartz and Pournaghdali (2020) conjectured that other people's TOTs may serve as a potential source of information for one's own TOT. They hypothesized that similarly to social contagion of false memories (Roediger et al., 2001), TOTs can be communicated to others. If this were the case, they asked, would a TOT reported by a confederate cause a TOT to be experienced by a genuine participant? In exploratory pilot studies run in our laboratory, though, repeated attempts to induce a TOT in a genuine participant, by asking one to three confederates around the table to report a TOT, have basically failed. First, a TOT was not more likely to be experienced by the participant when a given confederate reported a TOT than when no confederate reported one. Second, we observed that the genuine participant sometimes reported a TOT simultaneously with, or even before, the confederate, preventing a causal relationship to be established. In order to circumvent this problem, we asked the confederate to report a TOT very early in the trial. Still, no evidence of TOT contagion emerged. Inducing socially shared TOTs in the laboratory turned out to be more complex than expected.

On the surface, that several persons could experience a TOT within a small group may suggest that TOT states, as false memories, can be transmitted from one person to another. But just as false memories implanted by a confederate, social contagion comes from *outside* the person. Underneath the surface, we hypothesized that an *internal* factor may be involved, metacognitive appraisal of group recall efficiency. There should be more instances of remembering in several heads than in one. From this, we conjectured that people remembering together entertain—consciously or unconsciously—the inference that successful retrieval is more likely in group recall than in a single-person recall situation. Such a metacognitive appraisal may drive a stronger feeling of closeness with the target word and of recall imminence, precipitating one (or more people) into a TOT state. This reasoning fitted nicely with Schwartz and Metcalfe's (2011) metacognitive, inferential account of the TOT phenomenon. From their metacognitive standpoint, a TOT is conceived “not as a marker of failed retrieval, but as a premonition of future recall” (p. 744). If various situational clues for retrieval success build up to the point of exceeding a criterion, then the metacognitive monitoring system delivers a near-retrieval “warning” (TOT) signal. Such clues include cue familiarity (Metcalfe et al., 1993), target-related information (Schwartz and Smith, 1997), as well as cue-induced emotional arousal (Schwartz, 2010). Manipulating cue familiarity or the amount of target-related information has been shown to selectively affect TOTs without affecting retrievability, supporting a functional dissociation between memory retrieval mechanisms on the one hand and metacognitive monitoring mechanisms on the other. We contended that group recall, by magnifying the inference that targets will be successfully retrieved, acts as yet another situational clue, prompting the metacognitive monitoring system to launch more TOT signals than it would in a single-person recall situation.

### The Present Study

Clark et al. (2000) have established an important distinction between “group recall” and “collaborative recall.” Group recall involves individuals remembering independently from each other and then pooling their output, while collaborative recall involves social interactions: sharing cues, engaging discussions, and making a decision about what constitutes the correct answer, by consensus or by taking a majority vote. Group recall as defined by Clark et al. (2000) was the ideal experimental setting to isolate the metacognitive appraisal factor from the social contagion factor in socially shared TOTs.

To induce socially shared TOTs in the laboratory, we asked small groups of four (genuine) participants to think aloud and to cross-cue each other in order to recall answers to general knowledge questions. Although participants were instructed “to collaborate” together, severe constraints were imposed upon collaborative recall. First, each general knowledge question was presented for only 15 s, including the time for the experimenter to read the question, leaving little room for participants to think about cues and to actually communicate them to others. Second, participants were asked to refrain from revealing the right answer, as well as from telling others when they were experiencing a TOT. Because participants in small groups were (for a large part) recalling independently from each other, rather than engaging into discussions to come out with one agreed-upon answer, in the present study, “group recall” as defined by Clark et al. (2000), not “collaborative recall,” was assumed to take place.

If social contagion is the main factor involved in socially shared TOTs, then preventing explicit communication of TOT states in small groups should mask—at least to some extent—its effects. However, beyond social contagion, a primary determinant of socially shared TOTs could be metacognitive appraisal of group recall efficiency. Unlike social contagion, such an appraisal does not require that people interact with each other. Thus, preventing explicit communication of TOT states allowed us to test more directly our metacognitive appraisal hypothesis. In fact, in the group recall condition, all participants remained silent during most (71%) of the trials, no collaborative recall happening at all. Then, one may ask, what makes the group recall condition different from the individual recall condition? As hypothesized, only participants in small groups should entertain the inference that group recall is more efficient than individual recall. Their past collective remembering experience provided ample grounds to support this metacognitive appraisal of group recall efficiency. Control participants were tested alone with an identical set of general knowledge questions and received the same set of instructions: To think aloud and to cue themselves during recall, as well as to refrain from revealing the right answer or from telling the experimenter when they were experiencing a TOT.

If the metacognitive monitoring system indeed tracks clues that the target word will be successfully retrieved, as posited in Schwartz and Metcalfe's (2011) inferential view, then the probability of experiencing a TOT should be shown to markedly increase from the individual to the group recall conditions. Moreover, if group recall selectively increases TOTs without affecting correct recall, as it is the case for other social factors, social pressure (demand characteristics; Widner et al., 1996) and social stress (James et al., 2018; Schmank and James, 2020), then it would add to the accumulating evidence that the TOT phenomenon is dissociable from the retrieval process.

## Methods

### Participants

Forty-eight English-speaking Laurentian University students, 12 men and 36 women, participated in exchange for course credits. None reported being fluent in another language. They were recruited *via* SONA and, upon enrollment in the study, were randomly assigned to the individual (*n* = 24) or to the group (*n* = 24) recall condition. There were six small groups, each composed of four participants. The mean age of participants did not vary between those assigned to the individual recall condition (*M* = 25.08; *SD* = 9.45) and those assigned to the group recall condition (*M* = 25.92; *SD* = 9.85), *t*_(46)_ = −0.30, *p* = 0.77. They were informed that the study was about memory for general knowledge questions and that their participation involved undertaking two memory tests. Participants in the group recall condition were additionally informed that they would have to collaborate with three other participants in order to recall the right answers.

### Materials

The 80 general knowledge questions used in the present study (Supplementary Material) were taken from, adapted from, or written in the same form as, the Nelson–Narens set of 300 normalized questions (Nelson and Narens, 1980). Some questions were directly taken, or slightly reformulated, from the norms (e.g., *What is the last name of the scientist who formulated the theory of relativity?—*Einstein). Because our sample was composed of Canadian participants, American-specific questions (e.g., *What is the name of the ship that carried the Pilgrims to America in 1620?—*Mayflower) were adapted to become Canadian-specific questions (e.g., *What is the name of the ship depicted on the Canadian dime?—*Bluenose). And because most of our participants were born 15 years after the publication of the Nelson–Narens norms, questions about television shows, movie actors/characters, sports, literature, and music that were likely to be unknown by our participants (e.g., *What is the name of the Lone Ranger's Indian sidekick?*—Tonto) were adapted to the new generation (e.g., *What is the name of the gold-plated, humanoid robot in Star Wars?*—C-3PO). Finally, new questions were written in the same form as the Nelson–Narens normative questions (e.g., *What is the name of the curved stick that returns to you once thrown?—*boomerang). The knowledge domains covered by the 80 questions were arts, major events, famous people, geography, history, literature, music, nature, politics, science, sports, food, games and toys, television, and movies.

### Procedure

The experiment comprised two distinct phases, a free recall task, followed by a recognition task. In the free recall task, general knowledge questions were presented to participants, who had 15 s to provide the answer. The following recognition task, a four-alternative, multiple-choice recognition test, served as the criterion task to validate reported TOTs. A “TOT” response in the free recall task for a target word correctly recognized in the criterion task was classified as a “positive” TOT; otherwise, it was classified as a “negative” TOT. Upon enrollment, we did not make participants aware that the “second memory test” was a multiple-choice test involving the same 80 general knowledge questions as those used in the first memory test (free recall), to prevent TOT judgments from being confused with feeling-of-knowing (FOK) judgments (the feeling to know the target word and to be able to recognize it among a list of words).

#### Free Recall Task

**Figure 1:**
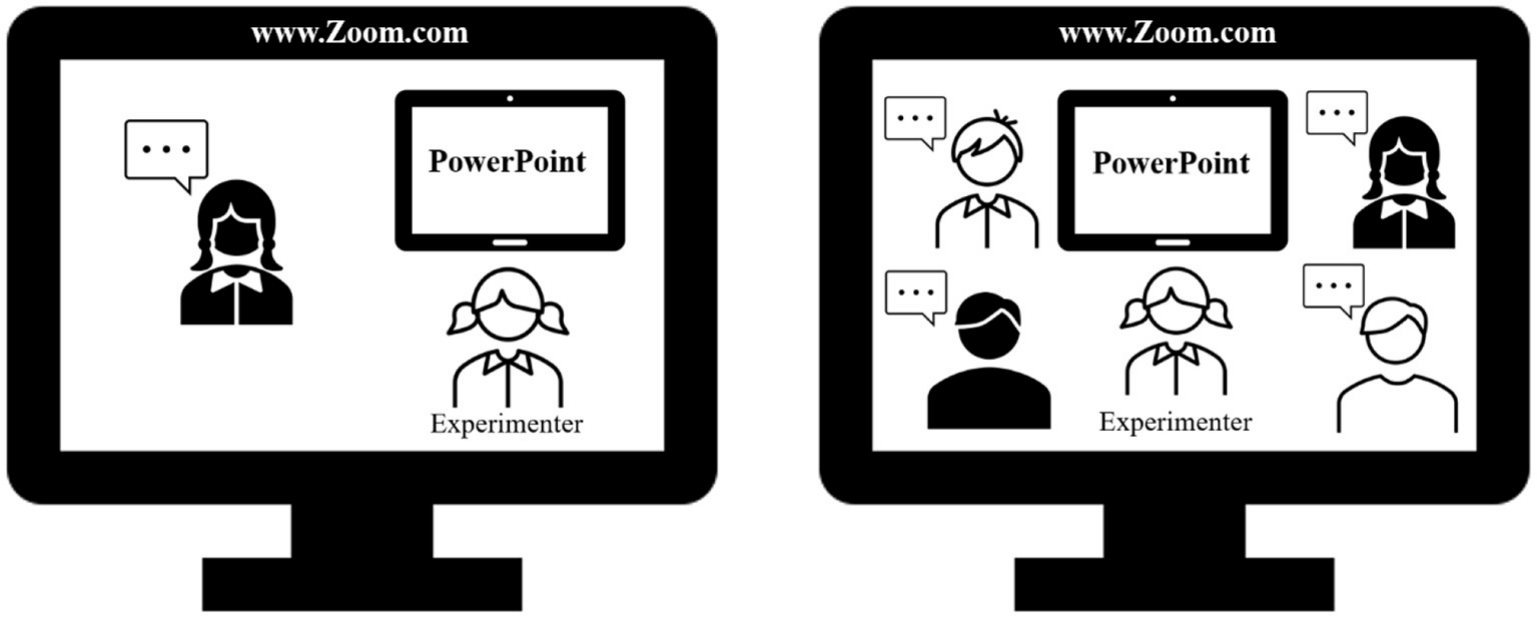
Sketch of the Zoom video call setting for the single-person (left) and the small group (right) recall conditions. Clip art is from Microsoft PowerPoint, version 16.50 (Microsoft Corporation).

Because the present study took place during the COVID-19 pandemic, access to campus laboratories was restricted and testing was conducted online. The experimenter hosted a Zoom video call session (see Figure 1) joined by one participant (individual recall condition) or by four participants (group recall condition). In both individual and group conditions, the experimenter and the participant(s) had their microphone and camera on. The experimenter shared her screen with a PowerPoint presentation, which served to present instructions and general knowledge questions. The text appeared in the Times New Roman font, in large yellow lettering on a black background. The written instructions were read by the experimenter (the text in brackets was presented to participants tested alone):

*In this experiment, you will be presented with 80 general knowledge questions. Your task is to collaborate with each other to recall the right answer. [Your task is to recall the right answer.] For each question, you will have 15 s to cross-cue each other [to cue yourself] by saying aloud everything that comes to your mind about the right answer. Please refrain from speaking out the right answer, keep it for yourself. After 15 s, you will hear a beep sound signaling the end of the “group recall” [recall] period and see a stop sign appear on the screen. Stop communicating [speaking] right away. If you do recall the answer, please check “Know” and write down the answer. If you do not know the answer, please check “Don't know.” If you feel quite sure you know the answer* and *you have a strong feeling that it is on the verge of coming back to you, then you are experiencing a “Tip-of-the-tongue” state. If so, please check “TOT” (Tip-of-the-tongue). Please do not check “TOT” simply because you feel quite sure you know the answer. In order to check “TOT,” you must* also *have a strong feeling that the known answer is on the verge of coming back to you (as if you were just about to spit it out). Please refrain from telling others [the experimenter] that you are experiencing a TOT, keep it for yourself*.

The instructions were followed by two trial runs. After the first trial run, participants in small groups were informed that they were allowed to continue collaborating with the others even if they had already found the right answer.

The 80 general knowledge questions were presented in random order, based on a randomization sub-routine implemented in PowerPoint. Each trial lasted 15 s: The question was displayed, read by the experimenter, and participants engaged in collaborative recall for the remaining time. At the end of the trial, a beep sound was heard and a stop sign was displayed. It prompted participants to privately fill out a *REDCap* response form. They were asked to choose among three options: “Know,” “Don't Know,” or “TOT.” By clicking “Know,” a blank text box was displayed in which the answer could be typed. The experimenter waited for all participants' forms to be filled out (about 10 s) before removing the question and starting the next trial. The free recall session lasted about 50 min.

#### Recognition Task

Before leaving the Zoom video call, each participant received a private link to a *Google Forms* “survey,” the four-alternative, multiple-choice recognition (criterion) test. The same 80 general knowledge questions were presented in random order, followed by a choice of four potential answers listed on the vertical axis without option letters (a, b, c, d). The three distractors were chosen as to be plausible alternatives (e.g., Bach, Mozart, and Schubert for target Beethoven). The position of the correct answer was counterbalanced. Participants were instructed to select the correct answer to the best of their knowledge, by relying only on their own memory, and to refrain from consulting Google to preserve the scientific integrity of the study. The selection was made by clicking a small circle beside each option.

## Results

In both experimental conditions, there were a total of 1920 trials (24 participants × 80 general knowledge questions). With regards to the group recall condition, the term “shared trial” refers to the presentation of a given general knowledge question to four participants. Thus, there were a total of 480 shared trials (six small groups × 80 general knowledge questions). The data from five shared trials were removed from the analyses because the correct answer was spoken out by a participant during the 15-s recall period (e.g., “His first name is Sidney” for target Crosby). Therefore, regarding small groups, the analyses were performed on 475 valid shared trials (475 × four participants = 1900 valid trials). The results of interest are presented in Table 1.

**Table 1:** Mean (standard deviation) percentages of correctly recalled items, incorrect and “Don't Know” responses, negative and positive TOT indexes, by recall condition (all trials).

	**Individual recall**	**Small group recall**	***t*_**(46)**_**	***p***	**Cohen's *d***	**95% CI (Cohen's *d*)**
Correct recall	65% (16%)	62% (14%)	0.76	0.45	–	–
Incorrect	6% (4%)	7% (3%)	−0.05	0.96	–	–
Don't know	25% (15%)	22% (11%)	−0.82	0.21	–	–
Negative (–) TOTs	0.2% (0.4%)	1% (3%)	−2.12	0.04	0.37	−0.43 – 1.18
**Positive (+) TOTs**						
Of all items	3% (3%)	8% (5%)	−4.10	<0.001	1.21	0.34 – 2.08
Of known	4% (7%)	12% (8%)	−3.42	<0.01	1.06	0.21 – 1.92
Of unknown	7% (7%)	20% (10%)	−4.79	<0.001	1.51	0.60 – 2.41

### Correct Recall

“Know” responses were categorized by hand as correct or incorrect (commission errors). Responses with spelling errors were categorized as correct as much as pronunciation was fairly close (e.g., *bumarang* for *b*oomerang). Correct recall did not differ between the individual recall (*M* = 65%; *SD* = 16%) and the group recall (*M* = 62%; *SD* = 14%) conditions, *t*_(46)_ = 0.76, *p* = 0.45. This result suggests that random sampling enabled equally distributed general knowledgeability among samples. More critically, this finding indicates that the experimental manipulation did not affect target retrievability.

### Incorrect and “Don't Know” Responses

Incorrect responses (commission errors) did not differ between the individual recall (*M* = 6%; *SD* = 4%) and the small group recall (*M* = 7%; *SD* = 3%) conditions, *t*_(46)_ = −0.05, *p* = 0.96. As well, “Don't Know” responses (omission errors) did not differ between the individual recall (*M* = 25%; *SD* = 15%) and the small group recall (*M* = 22%; *SD* = 11%) conditions, *t*_(46)_ = 0.82, *p* = 0.21. These results indicate that when recall failed, the number of trials available to report a TOT was similar in both experimental conditions.

### Negative TOTs

In most studies, so-called “negative” TOTs (–TOTs), that is, reported TOTs that are not considered as accurate because the target word is not recognized in the criterion test, are either discarded from the analyses or, based on the assumption (from the metacognitive standpoint) that TOTs are not always reflective of a memory trace, are considered as legitimate and therefore added to the pool of “positive” TOTs (+TOTs). In the present study, because the experimental manipulation was unprecedented, –TOTs were retained and analyzed separately from +TOTs. In the individual recall condition, there were a total of three –TOTs made by three participants while by contrast in the group recall condition, there were a total of 28 –TOTs made by seven participants (one participant made 11). Thus, –TOTs were rare but disproportionately higher in small groups. Expressed as a proportion of all trials, –TOTs were five time more likely to occur in small groups (*M* = 1%; *SD* = 3%) than in individuals (*M* = 0.2%, *SD* = 0.4%), *t*_(46)_ = −2.12, *p* = 0.04, *d* = 0.37, 95% CI (−0.43–1.18).

This finding is puzzling as we are not aware of any other study having reported an experimental manipulation affecting –TOTs. At first glance, it may reflect participants' propensity to claim being in a TOT state in social settings. However, because TOTs were covertly reported here, this interpretation is hazardous and would deserve to be tested more stringently by comparing covert to overt TOT reports in small groups. According to Brown (2012), negative TOTs are driven either by an increase in item difficulty or by the adoption of a more liberal criterion to report TOTs. He also mentions the negative correlation found by Abrams et al. (2003) between vocabulary knowledge and negative TOTs. Because correct recall did not differ between our experimental conditions, it is reasonable to assume that both item difficulty and vocabulary knowledge were comparable across samples. Therefore, at this point, we tentatively consider extraneous –TOTs in the group recall condition to derive from the adoption of a more liberal criterion to report TOTs, understood as an upward shift toward the criterion set by the metacognitive monitoring mechanism to launch a TOT signal in Schwartz and Metcalfe's (2011) view.

### Positive TOTs

A TOT was considered as “positive” (+TOT) when the target word was recognized in the four-alternative, multiple-choice (criterion) test. If TOTs are accurate predictors of retrieval success, they cannot always be accurate, but must be more accurate than non-TOT (incorrect and “Don't Know”) responses. In the present study, the target word recognition rate was higher following a TOT than following a non-TOT response in both individual recall (91 and 74%, respectively), *t*_(16)_ = 2.46, *p* = 0.02, *d* = 0.85, 95% CI (0.02–1.69), and group recall (89 and 74%, respectively), *t*_(23)_ = 3.45, *p* < 0.01, *d* = 1.03, 95% CI (0.18–1.89). If TOTs reported by participants in small groups were contaminated by high FOK judgments, then the recognition rate after a TOT should be higher in the group recall condition. However, the recognition rate following a TOT was not affected by the experimental manipulation [*t*_(39)_ = −0.30, *p* = 0.77], ruling out contamination by high FOK judgments as a viable alternative explanation for increased TOTs in the group recall condition.

In the field, a TOT is conceived either as a memory retrieval monitoring signal (metacognitive view) or as a side effect of the lexical retrieval process (psycholinguistic view). In the metacognitive view, +TOTs are often reported as a proportion of “unknown” items, using the term [incorrect responses + “Don't Know” responses + positive TOTs + negative TOTs] as the denominator, an index initially proposed by Brown (1991) to control for item difficulty [see also Burke et al. (1991)]. Alternatively, in the psycholinguistic view, based on the assumption that a TOT followed by a correct word recognition reflects access to the semantic representation of the target word (although the “second step” of the retrieval process, access to its phonological form, has allegedly failed), Gollan and Brown (2006) have proposed to report +TOTs as a proportion of “known” items, using the term (correctly recalled items + positive TOTs) as the denominator. Schwartz and Pournaghdali (2020) recommend reporting TOTs both ways and to discontinue the “highly problematic” practice of reporting TOTs as a proportion of all items. Nevertheless, because correct recall did not differ between our experimental conditions (mitigating potential index bias) and for sake of completeness, all three +TOT indexes are reported here [see also James et al. (2018) and Schmank and James (2020)].

All 24 participants in the group recall condition reported at least one +TOT, while 17 participants out of 24 reported at least one +TOT in the individual recall condition. Overall, 145 +TOTs were reported by participants tested in small groups and 51 +TOTs were reported by participants tested alone. For +TOTs expressed as a proportion of all 80 items, there was a 167% increase from the individual recall (*M* = 3%; *SD* = 3%) to the group recall (*M* = 8%; *SD* = 4%) conditions, *t*_(46)_ = −4.10, *p* < 0.001, *d* = 1.21, 95% CI (0.34–2.08). Note that this effect was significant despite the fact that the correct recall rate in the present study (about 64%) was considerably higher than the average rate (15%) reported in the field [115 TOT experiments or conditions, from 47 studies published between 1966 and 2010; see Brown (2012), Table 3.2]. For TOTs expressed as a proportion of known items, there was a 200% increase from the individual recall (*M* = 4%; *SD* = 7%) to the group recall (*M* = 12%; *SD* = 8%) conditions, *t*_(46)_ = −3.42, *p* < 0.01, *d* = 1.06, 95% CI (0.21–1.92). Finally, for +TOTs expressed as a proportion of unknown items, there was a 186% increase from the individual recall (*M* = 7%; *SD* = 7%) to the group recall (*M* = 20%; *SD* = 10%) conditions, *t*_(46)_ = −4.79, *p* < 0.001, *d* = 1.51, 95% CI (0.60–2.41). Thus, irrespective of the chosen +TOT index, the group recall condition generated more feelings of imminent recall.

### Common TOTs

The most obvious evidence for social contagion is obtained when, in a small group, several participants report a TOT for the same target. Out of 475 valid shared trials in small groups, 24 (5%), distributed across 21 different items, elicited TOTs in more than one participant: fourteen shared trials with 2 +TOTs, four with 3 +TOTs, four with 1 +TOT and 1 –TOTs, one with 2 +TOTs and 1 –TOT, and one with 2 –TOTs. Furthermore, out of 145 +TOTs observed in small groups, 46 (32%) were concurrently reported and out of 28 –TOTs, 7 (25%) were concurrently reported. Given that common TOTs represent the “signature” of social contagion, if social contagion is the main factor involved in the observed effect, then the effect should vanish when common TOTs are removed from the analyses. However, removing this data subset, that is, all trials (24 shared trials × 4 participants = 96 trials) for which more than one TOT—either positive or negative—were reported (53 TOTs/173; 31%), did not change the basic pattern of results (see Table 2). Correct recall was still unaffected by the experimental manipulation (*t* < 1) and all three +TOT indexes were still significantly higher in the group recall condition (Cohen's *d'*s > 0.71 < 0.99). These results suggest that social contagion, as indexed by common TOTs, was not the main factor involved in the additional TOTs observed in small groups.

**Table 2:** Mean (standard deviation) percentages of correctly recalled items, incorrect and “Don't Know” responses, negative and positive TOT indexes, by recall condition, after removing trials with common TOTs (31% of TOTs).

	**Individual recall**	**Small group recall**	***t*_**(46)**_**	***p***	**Cohen's *d***	**95% CI (Cohen's *d*)**
Correct recall	65% (16%)	64% (14%)	0.29	0.78	–	–
Incorrect	6% (4%)	7% (3%)	−0.24	0.81	–	–
Don't know	25% (15%)	22% (11%)	0.79	0.43	–	–
Negative (–) TOTs	0.2% (0.4%)	1% (2%)	−2.07	0.04	0.55	−0.26 – 1.37
**Positive (+) TOTs**						
Of all items	3% (3%)	6% (4%)	−2.54	0.01	0.85	0.01 – 1.68
Of known	4% (7%)	9% (7%)	−2.16	0.04	0.71	−0.11 – 1.54
Of unknown	7% (7%)	15% (9%)	−2.98	<0.01	0.99	0.14 – 1.84

### Verbal Exchanges

Although common TOTs are indicative of social contagion, it may still be the case that, in small groups, a TOT reported by a single participant was caused by social contagion. Indeed, the participant who is the source of contagion may have resolved their TOT within the 15-s free recall period or may have changed their mind and reported an incorrect or a “Don't Know” response instead of a “TOT” response (to substantiate this suspicion, two participants added the acronym “TOT” within parentheses after their correct response). To explore this issue, we have examined the verbal exchanges recorded during the 15-s free recall period. Of note, the 15-s period included the time for the experimenter to read the question, leaving little room for participants to think about cues and to actually communicate them to others. As a consequence, for the vast majority of valid shared trials (336/475; 71%), participants remained silent. Verbal exchanges actually occurred only on every third shared trial, on average (139/475; 29%). Given such a ratio, it is not surprising that only 21% of TOTs observed in small groups (37/173) occurred following verbal exchanges.

Overall, there were 166 verbal exchanges distributed across 139 of the 475 valid shared trials: 114 shared trials with one verbal exchange, 23 with two verbal exchanges, and two with three verbal exchanges. All verbal exchanges are considered here, including those coming from participants who provided the correct answer, as they were allowed to provide cues after having found the right answer (note that the correct answer may also have been found *after* providing cues). Verbal exchanges were divided into 10 categories: first letter correct (e.g., *It starts with an I* for target inuksuk); first letter incorrect; asking for the first letter or for a hint; semantic cue (e.g., *He has a winery in Niagara-on-the-Lake* for target Gretzky); phonological cue (e.g., *It rhymes with linguini* for target Houdini); indirect TOT (e.g., *I know this one!*); “blocker” (e.g., *It's not R2-D2* for target C-3PO); exclamation (e.g., *Oh my goodness!*); don't know (e.g., *I have no idea*); and commentary (e.g., *I love this place* for target Venice). Some verbal exchanges included interjections from more than one category. Table 3 reports correct recall, incorrect and “Don't Know” responses, as well as positive and negative TOT rates following verbal exchanges from a single category and from two or three mixed categories. The verbal exchange categories are listed from the most common (appeared in highest frequency of shared trials) to the least common.

**Table 3:** Correctly recalled items, incorrect and “Don't Know” responses, positive and negative TOTs, expressed as a frequency (percentage) of trials, by verbal exchange category.

**Verbal exchange category**	***n* shared trials**	***n* trials**	**Correct recall**	**Incorrect**	**“Don't know”**	**+TOT**	**–TOT**
Semantic cue	33	132	88 (67%)	9 (7%)	26 (20%)	9 (7%)	0 (0%)
First letter correct	24	96	74 (77%)	6 (6%)	14 (15%)	2 (2%)	0 (0%)
Two mixed categories	23	92	59 (64%)	6 (7%)	17 (18%)	9 (10%)	1 (1%)
Commentary	21	84	49 (58%)	7 (8%)	20 (24%)	8 (10%)	0 (0%)
Don't know	10	40	10 (25%)	3 (8%)	24 (60%)	2 (5%)	1 (3%)
Asking for first letter or hints	9	36	24 (67%)	2 (5%)	9 (25%)	1 (3%)	0 (0%)
Phonological cue	6	24	19 (79%)	2 (8%)	3 (13%)	0 (0%)	0 (0%)
Exclamation	4	16	11 (69%)	1 (6%)	1 (6%)	3 (19%)	0 (0%)
Indirect TOT	3	12	10 (83%)	1 (8%)	1 (8%)	0 (0%)	0 (0%)
“Blocker”	3	12	7 (58%)	1 (8%)	3 (25%)	1 (8%)	0 (0%)
Three mixed categories	2	8	4 (50%)	0 (0%)	4 (50%)	0 (0%)	0 (0%)
First letter incorrect	1	4	1 (25%)	3 (75%)	0 (0%)	0 (0%)	0 (0%)
TOTAL	139	556	356 (64%)	41 (7%)	122 (22%)	35 (6%)	2 (0.4%)

Although some categories of verbal exchanges occurred in very few shared trials, preventing quantitative analyses to be performed, we bring forth some observations. First, whether or not participants who provided the (correct) first letter of the sought-for word experienced a TOT, they nevertheless exposed others to a preeminent feature of a TOT state (Brown and McNeill, 1966), as well as evoking the popular “alphabet-scan” TOT resolution strategy. It does not seem, though, that such exposure was effective in inducing TOTs, as only 6% of TOTs following verbal exchanges (2/35 trials) were reported in that category. Second, the interjections (e.g., *I have no idea*) that also served as a response option (“Don't Know”) gave rise to a substantial increase of “Don't Know” responses accompanied by a substantial decrease of correctly recalled items. Did social conformity take place following this specific category of verbal exchanges? Third, 26% of +TOTs following verbal exchanges (9/35) were reported after a semantic cue. This observation is consonant with Schwartz and Smith's (1997) finding that the more target-related information is provided, the more TOTs are reported. Fourth, even if commentaries were provided in 15% of shared trials with verbal exchanges (21/139), 23% of +TOTs following verbal exchanges (8/35) were reported after commentaries. The +TOT rate was also disproportionately high after an exclamation, but this observation should be taken with caution because it is based on only four shared trials. Still, 31% of +TOTs (11/35) following verbal exchanges were reported after either a commentary or an exclamation. Fifth, 26% of +TOTs following a verbal exchange (9/35) occurred in the small portion (17%) of shared trials comprising a mix of two or three categories (23 shared trials/139). This observation suggests that the *number* of verbal exchanges may be as crucial as the contents of verbal exchanges to induce social contagion of TOTs.

Some verbal exchanges were followed by more than one TOT. Commentaries were followed by two +TOTs (2 shared trials) or three +TOTs (1 shared trial), while mixes of two verbal exchanges were followed by two +TOTs (1 shared trial) or three +TOTs (1 shared trial). These observations underline the potential role of commentaries, as well as of verbal exchanges from mixed categories, in the social contagion of TOTs.

Verbal exchanges may potentially interfere with one's specific retrieval strategy, as posited in the *collaborative interference effec*t (Weldon and Bellinger, 1997). One fellow might be using the “alphabet-scan” TOT resolution strategy, while another is providing target-related information and still another is holding his breath, waiting for the target to pop into their mind. The well-known *irrelevant speech effect* (Salamé and Baddeley, 1982) might also have disruptive effects on memory retrieval. In addition, “blockers” spoken out by others (e.g., *It's not R2-D2* for target C-3PO) may as well interfere with one's retrieval process. If there is interference, then TOTs might be more frequent following verbal exchanges. To explore these issues, in the group recall condition, we have compared the data subset with verbal exchanges (139 valid shared trials × four participants = 556 trials) to the data subset without verbal exchanges (336 valid shared trials × four participants = 1,344 trials) on all dependent variables of interest (see Table 4). Note that reported proportions are based on available items over 80, that is, 23 items with verbal exchanges and 56 items without verbal exchanges, on average. Unlike what would be predicted by a collaborative interference effect or by an irrelevant speech effect, retrievability was not hindered by verbal exchanges. Neither correct recall nor incorrect/“Don't Know” responses were affected by verbal exchanges (all *t'*s <1). Critically, TOTs were not more frequent following verbal exchanges, irrespective of the chosen TOT index (all *t'*s <1). These results suggest that verbal exchanges as a whole did not increase TOT states in small groups.

**Table 4:** Mean (standard deviation) percentages of correctly recalled items, incorrect and “Don't Know” responses, negative and positive TOT indexes, for trials with and without verbal exchanges.

	**Only trials *without* verbal exchanges**	**Only trials *with* verbal exchanges**	***t*_**(46)**_**	***p***
Correct recall	63% (17%)	60% (19%)	0.52	0.60
Incorrect	6% (4%)	7% (6%)	−0.85	0.40
Don't Know	22% (13%)	25% (14%)	−0.69	0.49
Negative (–) TOTs	2% (3%)	0.8% (3%)	−1.07	0.29
**Positive (+) TOTs**				
Of all items	7% (5%)	7% (8%)	−0.04	0.97
Of known	12% (9%)	12% (15%)	−0.20	0.85
Of unknown	19% (15%)	14% (14%)	−1.07	0.29

Despite the fact that TOT rates did not differ between the data subset with verbal exchanges and the data subset without verbal exchanges, if social contagion induced by verbal exchanges is the main factor involved in the TOT increase from the individual recall to the group recall conditions, then removing the data subset with verbal exchanges from the small groups' whole data set should abolish the effect. However, as shown in Table 5, removing this data subset, that is, all trials with verbal exchanges (139 shared trials × four participants = 556 trials), which gave rise to 21% of TOTs observed in small groups (37/173), did not change the basic pattern of results. Correct recall was still unaffected by the experimental manipulation (*t* < 1) and all three +TOT indexes were still significantly higher in the group recall condition (Cohen's *d'*s > 0.97 < 1.03). These results suggest that social contagion, as induced by verbal exchanges, was not the main factor involved in the additional TOTs observed in the group recall condition.

**Table 5:** Mean (standard deviation) percentages of correctly recalled items, incorrect and “Don't Know” responses, negative and positive TOT indexes, by recall condition, after removing trials with verbal exchanges (21% of TOTs).

	**Individual recall**	**Small group recall**	***t*_**(46)**_**	***p***	**Cohen's *d***	**95% CI (Cohen's *d*)**
Correct recall	65% (16%)	63% (17%)	0.50	0.61	–	–
Incorrect	6% (4%)	6% (4%)	0.66	0.51	–	–
Don't Know	25% (15%)	22% (13%)	0.79	0.44	–	–
Negative (–) TOTs	0.2% (0.4%)	2% (3%)	−2.34	0.02	0.84	0.01 – 1.68
**Positive (+) TOT**						
Of all items	3% (3%)	7% (5%)	−3.27	<0.01	0.97	0.12 – 1.82
Of Known	4% (7%)	12% (9%)	−3.12	<0.01	0.99	0.14 – 1.84
Of Unknown	7% (7%)	19% (15%)	−3.34	<0.01	1.03	0.17 – 1.88

### Common TOTs and Verbal Exchanges

Neither removing trials with common TOTs nor removing trials with verbal exchanges from the analyses changed the basic pattern of results. But does removing both data subsets abolish the observed effects? Note that these two data subsets are not mutually exclusive. Out of 475 valid shared trials, 24 comprised common TOTs, 139 comprised verbal exchanges, but five comprised *both*. Therefore, removing both data subsets resulted in the removal of 158 shared trials (42%), instead of 163. The same holds for TOTs. Out of 173 TOTs (145 +TOTs and 28 –TOTs), 53 (31%) were concurrently reported, 37 (21%) were reported after verbal exchanges, but 12 (12 +TOTs and no –TOTs; 7%) were reported both concurrently and after verbal exchanges. Therefore, removing both data subsets resulted in the removal of 78 TOTs (45%), instead of 90. As shown in Table 6, removing both data subsets, that is, all trials with common TOTs and/or verbal exchanges (158 shared trials × four participants = 632 trials), did not change the basic pattern of results. Correct recall was still unaffected by the experimental manipulation (*t* < 1) and all three +TOT indexes were still significantly higher in the group recall condition (Cohen's *d'*s > 0.67 < 0.73). These findings suggest that a TOT was more likely to be reported in a small group than in a private situation, regardless of whether or not a fellow also experienced one and regardless of whether or not the memory quest generated verbal exchanges.

**Table 6:** Mean (standard deviation) percentages of correctly recalled items, incorrect and “Don't Know” responses, negative and positive TOT indexes, by recall condition, after removing trials with common TOT and/or verbal exchanges (45% of TOTs).

	**Individual recall**	**Small group recall**	***t*_**(46)**_**	***p***	**Cohen's *d***	**95% CI (Cohen's *d*)**
Correct recall	65% (16%)	65% (17%)	0.11	0.91	–	–
Incorrect	6% (4%)	6% (4%)	0.42	0.67	–	–
Don't Know	25% (15%)	22% (13%)	0.075	0.46	–	–
Negative (–) TOTs	0.2% (0.4%)	1% (2%)	−2.35	0.02	0.55	−0.26 – 1.37
**Positive (+) TOTs**						
Of all items	3% (3%)	6% (5%)	−2.35	0.02	0.73	−0.10 – 1.55
Of known	4% (7%)	9% (8%)	−2.05	0.04	0.67	−0.16 – 1.49
Of unknown	7% (7%)	15% (14%)	−2.26	0.03	0.72	−0.10 – 1.55

## Discussion

In the past 30 years, authors have emphasized the need to investigate the TOT phenomenon in social settings (Brown, 1991; Schwartz and Cleary, 2016; Schwartz and Pournaghdali, 2020). To our knowledge, the present study represents the first attempt to extend Brown and McNeill's (1966) classic TOT prospection paradigm to small groups. A survey conducted on our university campus provided anecdotal evidence that shared TOTs are occasionally experienced in small groups. Drawing upon previous studies showing external factors, social pressure and social stress, to increase TOTs, our primary interest lay on the potential social contagion of TOTs. However, in exploratory pilot studies, repeated attempts to induce TOTs in a genuine participant, by asking one to three confederates around the table to claim being in a TOT state, have basically failed. We then turned to another, *internal* factor possibly involved in socially shared TOTs, metacognitive appraisal of group recall efficiency. There should be more instances of remembering in several heads than in one. From this, we conjectured that people remembering together entertain—consciously or unconsciously—the inference that successful retrieval is more likely in group recall than in a single-person recall situation. Such a metacognitive appraisal may drive a stronger feeling of closeness with the target word and of recall imminence, precipitating one (or more people) into a TOT state.

In order to isolate the effects of metacognitive appraisal from social contagion effects, severe constraints were put on collaborative recall. Participants in small groups were asked to refrain from telling others when they were experiencing a TOT, and verbal exchanges were limited to <15 s. In fact, participants in small groups remained silent during most (71%) of the trials. Regardless of the chosen TOT index (as a proportion of all items, as a proportion of known items, or a proportion of unknown items), we found that more TOTs were experienced by participants remembering in small groups than by participants remembering alone. When only silent trials were included in the analyses, TOTs were still more frequent in small groups. Moreover, when only TOTs reported by one of the four participants for a given item (rejecting common TOTs as the “signature” of social contagion) were included in the analyses, the pattern of results remained unchanged. In fact, TOTs reported only on silent trials by only one of the four participants were still more frequent than all TOTs reported by participants remembering alone. Therefore, we think that metacognitive appraisal of group recall efficiency is particularly powerful to drive TOTs in social situations.

According to Schwartz and Metcalfe's (2011) metacognitive, inferential account of the TOT phenomenon, the memory retrieval monitoring system launches a near-retrieval “warning” (TOT) signal when situational clues fueling the inference that the target word will be successfully retrieved reach a criterion. We argue that group recall provides yet another clue fueling the inference that successful recall is imminent, provoking an upward shift toward the criterion set by the metacognitive monitoring system to launch TOT signals. Critically, single-recall individual factors such as cue familiarity and target-related information (Metcalfe et al., 1993; Schwartz and Smith, 1997), social factors such as social pressure and social stress (Widner et al., 1996; James et al., 2018; Schmank and James, 2020), as well as group recall (the present experiment), have been shown to selectively increase TOTs without affecting correct recall, supporting a functional dissociation between metacognitive mechanisms on the one hand, and memory retrieval mechanisms on the other.

### Limitations, Alternative Interpretations, and Future Directions

#### Online vs. Face-to-Face Setting

Because the present study took place during the COVID-19 pandemic, access to campus laboratories was restricted and testing was conducted online. Instead of showing up at the laboratory, participants showed up at a Zoom session held by the experimenter. It should be noted that testing took place in November and December of 2020, rather than at the start of the pandemic, mitigating the novelty effect of video conferencing. Participants were full-time university students enrolled in online-delivered classes for two months prior to the present study. Although no particular experimental bias could be evoked, we nevertheless cannot rule out the possibility that the observed effects are specific to an online setting. Therefore, the present findings need to be generalized to a traditional face-to-face setting, to be considered as truly representative of small group cognitive functioning.

#### Social Contagion

Needless to say, despite the fact that participants were prevented from telling others when they were experiencing a TOT, social contagion was still likely to happen. First, there are indirect ways of verbally communicating a TOT to others, for instance by claiming knowledgeability (e.g., *Oh my God, I know it!*), by adopting a particular tone of voice, or by speaking out “blockers.” Second, removing trials with verbal exchanges does not exhaust all sources of social contagion. Indeed, there are non-verbal ways of communicating a TOT state. Some participants let out sighs and displayed heavy breathing. Furthermore, facial expressions, best characterized as winces or grimaces, were also displayed. Finally, despite the fact that computer cameras were mostly focused on participants' faces and shoulders, hand gestures were occasionally seen (in addition to head movements and jaw openings). Although research has been conducted on the relationships between gestures and TOTs (Frick-Horbury and Guttentag, 1998; Beattie and Coughlan, 1999; Theocharopoulou et al., 2015; Pyers et al., 2021), this research aimed at determining the extent to which gestures contribute to resolve TOT states, not at understanding how people communicate TOTs to others nor how they detect TOTs in others. At this point, the contribution of sighs, heavy breathing, facial expressions, and gestures to social communication of TOT states remains unknown. However, if social contagion did occur through one, or several, of those channels in the present study, then removing from the analyses all trials with common TOTs should have considerably reduced its effects. Because TOTs were still more frequent in small groups than in individuals after the removal of this data subset, it is unlikely that social contagion could explain most of the variability in the observed effect.

However, removing common TOTs from the analyses only provides an indirect test of a social contagion account of the present results. A more direct test would be to compare the likelihood of two or more people having a TOT for the same item in the group recall condition compared to the individual recall condition^1^. One way to do this is to create virtual groups of four from the individual condition and look at whether the real groups of four had more TOTs in common than the virtual groups. Unfortunately, the probability of two participants having a TOT for the same item is conditional upon the number of TOTs reported by the four participants. If each of the four participants reports only one TOT, then the probability that two of them report a TOT for the same item (assuming each TOT to be independently reported) is given by

$$\begin{array}{l} p=1-\left(80/80\times 79/80\times 78/80\times 77/80\right)=0.07\;\left(7\%\right). \end{array}$$

However, if each of the four participants reports two TOTs, then the probability of a common TOT becomes

$$\begin{array}{l} p=1-\left(80/80\times 78/80\times 76/80\times 74/80\right)=0.14\;\left(14\%\right). \end{array}$$

And if each participant reports six TOTs, then the probability is even more inflated:

$$\begin{array}{l} p=1-\left(80/80\times 74/80\times 68/80\times 62/80\right)=0.39\;\left(39\%\right). \end{array}$$

Because positive TOTs were 2.8 times more frequently reported in the group recall condition (*n* = 145; average of six TOTs per participant) than in the individual recall condition (*n* = 51; average of two TOTs per participant), directly comparing the likelihood of two people having a TOT for the same item between the real and the virtual groups would introduce a bias, with common TOTs being 25% more likely to occur in the real groups. An alternative way to conduct the same analysis is to create virtual groups from the group recall condition (one virtual group being composed of four participants from four *different* real groups) and look at whether the real groups of four had more TOTs in common than the virtual groups. Given fairly more balanced probabilities of a common TOT between the real and the virtual groups, more common TOTs in the real groups would be indicative of social contagion. The data of interest are presented in Table 7. As can be seen, the number of common TOTs is quite similar between the virtual and the real groups, further ruling out social contagion as a viable alternative explanation for the present results.

**Table 7:** Frequency of common positive TOTs in the six real groups (group recall condition), as well as in the six virtual groups (each composed of four participants from different real groups).

	**Real groups**			**Virtual groups**	
	**2 common TOTs**	**3 common TOTs**		**2 common TOTs**	**3 common TOTs**
	3	0		1	0
	3	0		2	0
	0	1		3	0
	4	0		4	0
	2	0		4	1
	3	3		1	0
Total	15	4	Total	15	1

In fact, item-specific factors, either linguistic (e.g., phonological neighborhood density, frequency per million words) or idiosyncratic, alternately could account for common TOTs observed in the group recall condition. Common TOTs were distributed across 17 different items in the real groups, while in the virtual groups, they were distributed across 16 different items. Out of the 17 common TOT items in the real groups, 11 (65%) were also common TOT items in the virtual groups, including one item (*inuksuk*) for which three participants shared a TOT in one real group as well as in one virtual group. We also examined items that elicited a TOT in more than one participant in the individual recall condition. Out of 80 items, seven elicited TOTs in more than one participant tested alone. Over a total of 51 TOTs in the individual recall condition, 22 (43%) were distributed across these seven items. Moreover, out of these 22 TOTs, 15 (68%) were distributed across four of the 17 common TOT items in the group recall condition (*Bluenose, Cherry, inuksuk*, and *Van Gogh*).

#### Social Contagion Through “Warm Glow”

Although we consider to have made a strong case against a social contagion account of the present findings, it does not mean that TOTs are not contagious. That other people's TOTs may be a potential source of information for one's own TOTs (Schwartz and Pournaghdali, 2020) is still a plausible hypothesis. But what mechanism would underlie TOT contagion? Emotional contagion (Hatfield et al., 1993) might be a good prospect. Schwartz and Pournaghdali (2020) conjectured that the so-called “warm glow”—a positive feeling for the sought-for word experienced during a TOT state (Cleary, 2019)—could be communicated to nearby people, precipitating them into a TOT state. If so, they speculated, maybe a TOT reported by a confederate could cause a TOT to be experienced by a genuine participant.

As previously mentioned, in exploratory pilot studies run in our laboratory, TOTs reported by confederates were not efficient at eliciting TOTs in a genuine participant. When a confederate artificially reports a TOT, no genuine mental state is associated with it. We think that Schwartz and Pournaghdali (2020) are right to conjecture that TOTs may potentially be transmitted to others through the “warm glow” mental state associated with it, but in our opinion, it would be mistaken to assume that a “warm glow” could emanate from an unfelt TOT reported by a confederate. Interestingly, Cleary (2019) speculated that the feeling of being in a TOT state is analogous to a familiarity feeling, itself associated with a positivity bias and a “warm glow.” In the present study, it was observed that 31% of TOTs following verbal exchanges involved commentaries (e.g., *It's my favorite team!*) and exclamations (e.g., *Oh my goodness!*). What do these two verbal exchange categories have in common? Both involve a personal, familiar, and emotional connection with the contents of the general knowledge question. For some participants, the target word evoked familiarity and it was communicated to others through vibrant emotional verbal utterances. We suspect that people who communicate familiarity for a sought-for word through emotionally-charged commentaries or exclamations may potentially convey a “warm glow” that is likely to be attributed to a TOT state by nearby people. Consequently, we think that the most promising way to induce social contagion of TOTs in the laboratory is to ask confederates not to report TOTs, but rather to lively communicate familiarity for preselected targets. Actually, in the same experiment, a confederate with good acting skills could be asked to simply report a TOT for some preselected targets (no “warm glow”), and lively communicate familiarity (“warm glow”) without reporting a TOT at all, for some others. We would predict genuine participants to experience more TOTs in the latter condition. Such an experiment only requires one confederate and one genuine participant, but it can also be extended to a small group composed of one confederate and three genuine participants. The latter condition should generate even more TOTs because two potential factors underlying socially shared TOTs—social contagion and metacognitive appraisal of group recall efficiency—would then be combined.

#### Demand Characteristics

Because TOTs are rare events, in laboratory studies researchers tend to use relatively difficult items, in order to increase the potential number of unrecalled targets and hence to collect more TOTs. Schwartz (2002) noted that such a practice might also introduce subtle demand characteristics. He argued that reporting a TOT is a way to socially communicate that knowledge exists for the target when that knowledge cannot be demonstrated. Faced with difficult items, participants might report more TOTs in order to not appear less knowledgeable. Here, the correct recall rate (64%) was considerably higher than the average rate (15%) in the field (Brown, 2012), making it unlikely that items' difficulty level exerted pressure to report TOTs. Moreover, the correct recall rate did not differ between the two experimental conditions. Because items were not more difficult for participants tested in small groups, they had no more reasons than participants tested alone to communicate that knowledge existed for unrecalled targets. But there is another reason why participants tested in small groups would report more TOTs in order to not appear less knowledgeable. In addition to the experimenter, three other persons were present, so that in small groups it was allegedly more embarrassing to not provide the correct answer right away. However, in the present experimental setting, “Know” and “TOT” responses were covertly reported. Reporting TOTs in a private, written form is obviously not an effective way to communicate to others that knowledge existed for the unrecalled target, as only the experimenter had access to TOT responses, post-experimentally. Still, *negative* TOTs were more prevalent in small groups, a finding that may suggest that private reports do not preclude knowledge to be claimed.

In the present study, no experimenter-induced pressure to report TOTs was exerted on participants by telling them that the general knowledge questions were normatively easy (high demand characteristics), as Widner et al. (1996) did. It does not mean, though, that participants in small groups were not led to infer that the questions were easy. However, if they did make such an inference, it was not based on the easiness of the questions themselves (no instructions were provided to this end), but on the easiness with which a small group could recover answers to general knowledge questions compared to a person recalling alone. In other words, to reiterate our interpretation of the present findings, participants tested in small groups could have been led to infer that the likelihood of successful recall was substantially higher in four heads than in one.

#### Social Conformity

In a classic series of experiments, Asch (1956) asked participants to make simple perceptual judgments, like judging which of three comparison lines is of the same length as that of a reference line. Before giving their response, participants watched six other participants (actually confederates) providing, one at a time, the same obviously erroneous answer. On these critical trials, 75% of participants conformed to the opinion of the majority at least once. Of note, Asch observed that conformity decreased significantly when confederates provided their answers aloud, but the genuine participant responded in writing. In the present experimental setting, four genuine participants were asked to provide answers to general knowledge questions. First, if a conformity effect did occur, it is hard to tell which response participants would have conformed to. Right answers were not spoken out and, critically, participants were instructed not to tell others when they were experiencing a TOT state. Second, all answers were provided in written form, making it unlikely that “TOT” responses were caused by social conformity. In fact, as previously pointed out, only “Don't Know” responses were likely to be induced by social conformity, through listening to verbal utterances such as *I have no idea!*

#### Social Loafing

People sometimes exert less effort to achieve a goal when they work in a group than when working alone. This classic phenomenon is known as *social loafing* (Karau and Williams, 1993). Admittedly, if participants exert less effort for recovering answers to general knowledge questions when remembering in small groups, then it could be expected that representational nodes would only be partially activated, resulting in more TOTs. However, partial activation would also hinder retrievability. Here, correct recall did not differ between the individual and the group recall conditions, ruling out social loafing as a viable alternative interpretation of the present findings.

#### Social Stress

It could be argued that in the group recall condition, the mere presence of three other persons induced social stress on the participants. Because social stress has been shown to induce TOTs (James et al., 2018; Schmank and James, 2020), it could possibly explain why more TOTs were observed in small groups. If social stress was indeed involved, and was felt more strongly shortly after being asked to interact with three strangers, then TOTs should be more frequent in the first half range of items. To test this possibility, we ran a 2 (recall condition) × 2 (item range) ANOVA on each positive TOT index. An interaction should be indicative of a social stress effect—a larger TOT rate difference between the two recall conditions in the first half than in the second half range of items. The results of interest are presented in Table 8. First, with regards to correct recall, there was no main effect of the recall condition [*F*_(1,92)_ = 1.00, *p* = 0.32], no main effect of the item range (*F* < 1), and no interaction (*F* < 1). Second, with regards to TOT rates, the analysis revealed a main effect of the recall condition for TOTs expressed as a proportion of all items in the relevant range [*F*_(1,92)_ = 28.82, *p* < 0.001, $\eta_{\text{p}}^{2}$ = 0.24], for TOTs expressed as a proportion of known items [*F*_(1,92)_ = 21.04, *p* < 0.001, $\eta_{\text{p}}^{2}$ = 0.19], and for TOTs expressed as a proportion of unknown items [*F*_(1,92)_ = 36.40, *p* < 0.001, $\eta_{\text{p}}^{2}$ = 0.28], but there was no significant main effect of the item range [*F* < 1; *F* < 1; *F*_(1,92)_ = 1.68, *p* = 0.20, respectively]. More critically, irrespective of the chosen TOT index, the recall condition × item range two-way interaction effect was not significant (all *F'*s < 1), ruling out social stress as a viable alternative interpretation of the present results.

**Table 8:** Mean (standard deviation) percentages of correctly recalled items and positive TOT indexes by experimental condition for the first and second half range of items.

	**First half range**		**Second half range**	
	**of items**		**of items**	
	**Individual recall**	**Small group recall**	**Individual recall**	**Small group recall**
Correct recall	67% (18%)	63% (16%)	64% (16%)	61% (14%)
**Positive (+) TOTs**				
Of all items	3% (3%)	7% (5%)	2% (4%)	8% (5%)
Of known	5% (6%)	11% (9%)	4% (7%)	12% (9%)
Of unknown	10% (8%)	21% (13%)	6% (8%)	19% (9%)

#### Functional Dissociations

The metacognitive account of the TOT phenomenon (Schwartz, 1999, 2002; Schwartz and Metcalfe, 2011) has been challenged. In contrast to earlier work (Metcalfe et al., 1993; Schwartz, 2010), subsequent studies failed to support the view that TOT states may be caused by cue familiarity or cue-induced emotional arousal (D'Angelo and Humphreys, 2012; Oliver et al., 2019). Before considering group recall as a retrieval-independent TOT-inducing factor, making a stringent, “double dissociation” case would be advisable. If the TOT phenomenon and the retrieval process are indeed functionally dissociable, then on the one hand, manipulating social variables should be shown to increase TOTs without affecting correct recall. Alternatively, on the other hand, manipulating memory variables should be shown to selectively affect correct recall without affecting TOTs.

A first step in that direction would be to manipulate group sizes. If socially shared TOTs are driven by metacognitive appraisal of group recall efficiency, then TOTs might potentially increase with the number of people (2, 4, 6, 8, etc.) sharing memories. Another option would be to manipulate the degree of prior relationship between members of small groups while fixing group size at four. Contrasting friends and strangers has been shown to influence the *collaborative inhibition effect* (Harris et al., 2012) and contrasting the social groups of speakers and listeners (e.g., Princeton vs. Yale students) has been shown to mediate the *socially shared retrieval-induced forgetting effect* (Coman and Hirst, 2015). The closer the relationship, the higher the likelihood of shared memories. For instance, a small group of close friends may have seen the same movie together, traveled to Europe together, or tried rock climbing for the first time together. If close friends of the same generation and culture assume to have been exposed to the same general knowledge over the years, then their subjective likelihood of successful retrieval for answers to general knowledge questions should increase when remembering together (*If I can't remember it, they will!*), compared to strangers. If this is the case, then in small groups, close friends should experience more TOTs than strangers, yet showing similar correct recall.

In turn, manipulating memory variables should be shown to selectively affect correct recall without affecting TOT rates. For instance, replicating the present experimental setting using a set of *difficult* general knowledge questions (e.g., 25% accuracy instead of 64%) should show a similar decrease in correct recall in the individual and the group recall conditions, but should not affect +TOT rates.

#### Social Functions of TOTs

In the field, the metacognitive research agenda has turned toward examining what functions TOTs may serve. It has been surmised that, as near-retrieval success “warning” signals, TOTs are an adaptive feature of the human cognitive system (Schwartz and Cleary, 2016). Feelings of imminent recall have been assumed to fuel epistemic curiosity and therefore to sustain efforts for recovering the sought-after word (Metcalfe et al., 2017, 2020). But TOTs could also fulfill social functions. As mentioned previously, according to Schwartz (2002), one function of TOTs is to socially communicate that knowledge exists for the target when that knowledge cannot be demonstrated. In the embarrassing situation of not remembering the name of an acquaintance approaching, one can claim to have a TOT for the person's name—rather than confessing to have forgotten it—to maintain a good relationship. Expanding on Schwartz's (2002) speculation that TOTs facilitate dyadic social interactions, we suspect TOTs to favor social bonding [see Bluck et al. (2005), for a similar functional view on autobiographical memories]. Hence, when someone in a small group has a TOT, peers sometimes claim to have a TOT themselves to express their empathy. Furthermore, in order to not offend the person struggling with a TOT, one could also, purposely, withhold the target word. Indeed, revealing the target word could spoil both the pleasure of collectively hunting for the target word and the reward associated with its recovery. In many cases, the person experiencing a TOT will insist not to be helped (*Wait, wait… don't tell me!*) in order to feel the great relief of recovering the target word. Claiming to be in a TOT state could keep the suspense alive! We chose to refer to the excitement shared by members of a small group anticipating the reward associated with successful recall as “the *Family Feud* happy feeling.” Some guests at social events will even teasingly fuel the socially shared TOT and trigger wild laughs by providing the most incongruous “blockers” (e.g., Céline Dion, rather than Kate Winslet, as the name of the actress playing *Rose* in the movie *Titanic*).

As noted by Schwartz (2002), although there is research about the detection of genuine and fake smiles, there has been no investigation on humans' abilities to detect genuine and fake TOTs. Furthermore, as it is the case for genuine smiles (Song et al., 2016), are people displaying genuine TOTs perceived as more prosocial than people displaying fake TOTs? And are people displaying fake TOTs in small groups perceived as more prosocial than people who abruptly dissolve socially shared TOTs by revealing the target word? What effects do congruous, but especially incongruous, “blockers,” have on social cohesion among members of a small group?

## Summary and Conclusion

This study represents the first attempt to extend Brown and McNeill's (1966) classic TOT prospection paradigm to small groups. Presented with general knowledge questions, participants tested in small groups reported more TOTs than participants tested alone. Critically, the experimental manipulation did not affect correct recall. Coupled with previous findings showing that social factors increase TOTs without affecting correct recall (Widner et al., 1996; James et al., 2018; Schmank and James, 2020), these data provide further support to the view that the TOT phenomenon is dissociable from the retrieval process (Schwartz, 1999, 2002; Schwartz and Metcalfe, 2011). Removing all trials with common TOTs and/or verbal exchanges from the analyses did not change the basic pattern of results, suggesting that social contagion was not the main factor involved in the observed effect. We argue that beyond social contagion, a powerful internal factor is involved, metacognitive appraisal of group recall efficiency. There should be more instances of remembering in several heads than in one. From this, we conjectured that people remembering together entertain—consciously or unconsciously—the inference that successful retrieval is more likely in group recall than in a single-person recall situation. Such a metacognitive appraisal may drive a stronger feeling of closeness with the target word and of recall imminence, precipitating one (or more people) into a TOT state. In line with Schwartz and Metcalfe's (2011) metacognitive, inferential account of the TOT phenomenon, we argue that similarly to other situational clues such as cue familiarity and target-related information, group recall magnifies the inference that the target word will be successfully retrieved, prompting the metacognitive monitoring system to launch more near-retrieval success “warning” (TOT) signals than in a single-person recall situation. Understanding the social dynamics of TOTs is still in its infancy, but it has the potential to advance our knowledge about how and why minds connect.

## Data Availability Statement

The raw data supporting the conclusions of this article will be made available by the authors, without undue reservation.

## Ethics Statement

The studies involving human participants were reviewed and approved by Laurentian University Psychology Department Research Ethics Board. The patients/participants provided their written informed consent to participate in this study.

## Author Contributions

LR conceptualized the study, designed the experiment, analyzed the data, and interpreted the results. NK adapted and wrote new general knowledge questions, conducted the experiment, and analyzed the data, in partial fulfillment of the requirements for her B.A. Honours thesis in Psychology. LR wrote the manuscript. Both authors approved the submitted manuscript.

## Conflict of Interest

The authors declare that the research was conducted in the absence of any commercial or financial relationships that could be construed as a potential conflict of interest.

## Footnotes

^1^We are thankful to an anonymous reviewer for suggesting this analysis.

## References

[B1] Abrams L. White K. K. Eitel S. L. (2003). Isolating phonological components that increase tip-of-the-tongue resolution. Mem. Cognit. 31, 1153–1162. 10.3758/BF03195798 15058676

[B2] Asch S. E. (1956). Studies of independence and conformity: I. A minority of one against a unanimous majority. Psychol. Monogr. Gen. Appl. 70, 1–70. 10.1037/h0093718

[B3] Beattie G. Coughlan J. (1999). An experimental investigation of the role of iconic gestures in lexical access using the tip-of-the-tongue phenomenon. Br. J. Psychol. 90, 35–56. 10.1348/000712699161251 10085545

[B4] Bluck S. Alea N. Habermas T. Rubin D. C. (2005). A TALE of three functions: the self-reported uses of autobiographical memory. Soc. Cogn. 23, 91–117. 10.1521/soco.23.1.91.5919826371517

[B5] Brown A. S. (1991). A review of the tip-of-the-tongue experience. Psychol. Bull. 109, 204–223. 10.1037/0033-2909.109.2.204 2034750

[B6] Brown A. S. (2012). The Tip of the Tongue State. New York, NY: Psychology Press. 10.4324/9780203582961

[B7] Brown R. McNeill D. (1966). The “tip of the tongue” phenomenon. J. Verb. Learn. Verb. Behav. 5, 325–337. 10.1016/S0022-5371(66)80040-3

[B8] Burke D. M. MacKay D. G. Worthley J. S. Wade E. (1991). On the tip of the tongue: what causes word finding failures in young and older adults? J. Mem. Lang. 30, 542–579. 10.1016/0749-596X(91)90026-G

[B9] Clark S. E. Hori A. Putnam A. Martin T. P. (2000). Group collaboration in recognition memory. J. Exp. Psychol. Learn. Mem. Cogn. 26, 1578–1588. 10.1037/0278-7393.26.6.1578 11185784

[B10] Cleary A. M. (2019). The biasing nature of the tip-of-the-tongue experience: when decisions bask in the glow of the tip-of-the-tongue state. J. Exp. Psychol. Gen. 148, 1178–1191. 10.1037/xge0000520 30489121

[B11] Coman A. Hirst W. (2015). Social identity and socially shared retrieval-induced forgetting: the effects of group membership. J. Exp. Psychol. Gen. 144, 717–722. 10.1037/xge0000077 25938179

[B12] Cuc A. Koppel J. Hirst W. (2007). Silence is not golden: a case for socially shared retrieval-induced forgetting. Psychol. Sci. 18, 727–733. 10.1111/j.1467-9280.2007.01967.x 17680945

[B13] D'Angelo M. C. Humphreys K. R. (2012). Emotional cues do not increase the likelihood of tip-of-the-tongue states. Mem. Cognit. 40, 1331–1338. 10.3758/s13421-012-0235-z 22833321

[B14] Frick-Horbury D. Guttentag R. E. (1998). The effects of restricting hand gesture production on lexical retrieval and free recall. Am. J. Psychol. 111, 43–62. 10.2307/1423536

[B15] Gollan T. H. Brown A. S. (2006). From tip-of-the-tongue (TOT) data to theoretical implications in two steps: when more TOTs means better retrieval. J. Exp. Psychol. Gen. 135, 462–483. 10.1037/0096-3445.135.3.462 16846276

[B16] Harris C. B. Barnier A. J. Sutton J. (2012). Shared encoding and the costs and benefits of collaborative recall. J. Exp. Psychol. Learn. Mem. Cogn. 39, 183–195. 10.1037/a0028906 22686851

[B17] Hatfield E. Cacioppo J. T. Rapson R. L. (1993). Emotional contagion. Curr. Dir. Psychol. Sci. 2, 96–99. 10.1111/1467-8721.ep10770953

[B18] James L. E. Schmank C. J. Castro N. Buchanan T. W. (2018). Tip of the tongue states increase under evaluative observation. J. Psycholinguist. Res. 47, 169–178. 10.1007/s10936-017-9524-9 29019103

[B19] Karau S. J. Williams K. D. (1993). Social loafing: a meta-analytic review and theoretical integration. J. Pers. Soc. Psychol. 65, 681–706. 10.1037/0022-3514.65.4.681

[B20] Metcalfe J. Schwartz B. L. Bloom P. A. (2017). The tip-of-the-tongue state and curiosity. Cogn. Res. Principl. Implicat. 2:31. 10.1186/s41235-017-0065-4 PMC5514176 28776003

[B21] Metcalfe J. Schwartz B. L. Eich T. S. (2020). Epistemic curiosity and the region of proximal learning. Curr. Opin. Behav. Sci. 35, 40–47. 10.1016/j.cobeha.2020.06.007 33709011 PMC7943031

[B22] Metcalfe J. Schwartz B. L. Joaquim S. G. (1993). The cue-familiarity heuristic in metacognition. J. Exp. Psychol. Learn. Mem. Cogn. 19, 851–861. 10.1037/0278-7393.19.4.851 8345327

[B23] Nelson T. O. Narens L. (1980). Norms of 300 general-information questions: accuracy of recall, latency of recall, and feeling-of-knowing ratings. J. Verb. Learn. Verb. Behav. 19, 338–368. 10.1016/S0022-5371(80)90266-2

[B24] Oliver L. K. Li T. Harley J. J. Humphreys K. R. (2019). Neither cue familiarity nor semantic cues increase the likelihood of repeating a tip-of-the-tongue state. Collabra Psychol. 5:28. 10.1525/collabra.200

[B25] Pyers J. E. Magid R. Gollan T. H. Emmorey K. (2021). Gesture helps, only if you need it: inhibiting gesture reduces tip-of-the-tongue resolution for those with weak short-term memory. Cogn. Sci. 45:e12914. 10.1111/cogs.12914 33389787 PMC7808404

[B26] Resnick L. B. Levine J. M. Teasley S. D. (1991). Perspectives on socially shared cognition. Washington, DC: Am. Psychol. Assoc. 10.1037/10096-000

[B27] Roediger H. L. III. Meade M. L. Bergman E. T. (2001). Social contagion of memory. Psychon. Bull. Rev. 8, 365–371. 10.3758/BF03196174 11495127

[B28] Salamé P. Baddeley A. (1982). Disruption of short-term memory by unattended speech: implications for the structure of working memory. J. Verb. Learn. Verb. Behav. 21, 150–164. 10.1016/S0022-5371(82)90521-7

[B29] Schmank C. J. James L. E. (2020). Adults of all ages experience increased tip-of-the-tongue states under ostensible evaluative observation. Aging Neuropsychol. Cogn. 27, 517–531. 10.1080/13825585.2019.1641177 31294648

[B30] Schwartz B. L. (1999). Sparkling at the end of the tongue: the etiology of tip-of-the-tongue phenomenology. Psychon. Bull. Rev. 6, 379–393. 10.3758/BF03210827 12198776

[B31] Schwartz B. L. (2002). Tip-of-the-Tongue States: Phenomenology, Mechanism, and Lexical Retrieval. Mahwah, NJ: Erlbaum. 10.4324/9781410604019

[B32] Schwartz B. L. (2010). The effects of emotion on tip-of-the-tongue states. Psychon. Bull. Rev. 17, 82–87. 10.3758/PBR.17.1.82 20081165

[B33] Schwartz B. L. Cleary A. M. (2016). Tip-of-the-tongue states, déjà vu experiences, and other odd metamemory experiences, in The Oxford Handbook of Metamemory, eds Dunlosky J. Tauber S. K. (Oxford, UK: Oxford University Press), 95–108. 10.1093/oxfordhb/9780199336746.013.5

[B34] Schwartz B. L. Metcalfe J. (2011). Tip-of-the-tongue (TOT) states: retrieval, behavior, and experience. Mem. Cognit. 39, 737–749. 10.3758/s13421-010-0066-8 21264637

[B35] Schwartz B. L. Pournaghdali A. (2020). Tip-of-the-tongue states: past and future, in Memory Quirks: The Study of Odd Phenomena in Memory. eds Cleary A. M. Schwartz B. L. (New York, NY: Routledge), 207–223. 10.4324/9780429264498-16

[B36] Schwartz B. L. Smith S. M. (1997). The retrieval of related information influences tip-of-the-tongue states. J. Mem. Lang. 36, 68–86. 10.1006/jmla.1996.2471

[B37] Song R. Over H. Carpenter M. (2016). Young children discriminate genuine from fake smiles and expect people displaying genuine smiles to be more prosocial. Evol. Hum. Behav. 37, 490–501. 10.1016/j.evolhumbehav.2016.05.002

[B38] Sutton J. Harris C. B. Keil P. G. Barnier A. J. (2010). The psychology of memory, extended cognition, and socially distributed remembering. Phenomenol. Cogn. Sci. 9, 521–560. 10.1007/s11097-010-9182-y

[B39] Theocharopoulou F. Cocks N. Pring T. Dipper L. T. (2015). TOT phenomena: gesture production in younger and older adults. Psychol. Aging. 30, 245–252. 10.1037/a0038913 25961875

[B40] Weldon M. S. Bellinger K. D. (1997). Collective memory: collaborative and individual processes in remembering. J. Exp. Psychol. Learn. Mem. Cogn. 23, 1160–1175. 10.1037/0278-7393.23.5.1160 9293627

[B41] Widner R. L. Smith S. M. Graziano W. G. (1996). The effects of demand characteristics on the reporting of tip-of-the-tongue and feeling-of-knowing states. Am. J. Psychol. 109, 525–538. 10.2307/1423392 8837408

