# Supplementary material for: Socially Shared Feelings of Imminent Recall: More Tip-of-the-Tongue States Are Experienced in Small Groups
Source: Front Psychol. 2021 Jul 16;12:704433. doi: 10.3389/fpsyg.2021.704433 (PMC8322979; doi:10.3389/fpsyg.2021.704433)
Supplement: Supplementary file 1 [file Table_1.docx]

***Supplementary Online Material***

Complete listing of the 80 general knowledge questions, target words, and alternative choice options.

[To accompany Rousseau, L., & Kashur, N. (2021). Socially shared feelings of imminent recall: More tip-of-the-tongue states are experienced in small groups. *Frontiers in Psychology*. <https://doi.org/10.3389/fpsyg.2021.704433>]

**80 general knowledge questions**

Target words (in alphabetical order), general knowledge questions, and alternative choice options. Target words preceded by an asterisk indicate an item taken from Nelson and Narens (1980); close adaptations are denoted by a number sign.

**Target word General knowledge question (alternative choice options)**

A&W™ What is the name of the fast-food restaurant chain that serves the Papa Burger™ and the Mama Burger™? (Burger King™, McDonald’s™, Wendy’s™)

Africa On which continent is Congo located? (Asia, Europe, South America)

Angry Birds™ In which popular video game do you attack pigs? (Candy Crush™, Minecraft™, Pokémon Go)

*Armstrong What is the last name of the first person to step foot on the Moon? (Aldrin, Gagarin, Glenn)

Astronomy What natural science studies planets, stars, and galaxies? (Earth science, Meteorology, Physics)

Audi Which luxury car brand has a four-ring logo? (BMW, Mercedes, Porsche)

Beatles What is the name of the British musical band that recorded the songs “Hey Jude” and “Let It Be”? (Led Zeppelin, Pink Floyd, Rolling Stones)

BeaverTails™ What is the name of the iconic Canadian pastry, hand stretched and topped with a choice of sweet condiments and confections? (Bear Claw, S’more, Sweet Bannock)

Beethoven Who wrote the famous Fifth Symphony? (Bach, Mozart, Schubert)

Bermuda Triangle What is the name of the region where a number of ships and aircrafts are said to have mysteriously disappeared? (Area 51, Easter Island, Stonehenge)

Bieber What is the last name of the Canadian male singer who was discovered on YouTube in his youth? (Drake, Mars, Weeknd)

Big Ben What nickname was given to the giant bell in downtown London, England? (London Eye, Piccadilly Circus, Square Mile)

Bin Laden What is the last name of the al-Qaeda leader who ordered the September 11, 2001, terrorist attacks? (Gaddafi, Hussein, Zarqawi)

Bluenose What is the name of the ship depicted on the Canadian dime? (Marco Polo, Royal William, W. D. Lawrence)

#Bond What is the last name of the British secret agent character, also known as “007”? (Bourne, Fleming, Powers)

Boomerang What is the name of the curved stick that returns to you once thrown? (Frisbee, Slingshot, Yo-yo)

Brady What is the last name of the New England Patriots’ quarterback, six-time Super Bowl champion? (Bledsoe, Manning, Wilson)

Bublé What is the last name of the Canadian singer who recorded the song “Haven’t Met You Yet” and released a very popular Christmas album? (Adams, Mendes, Sexsmith)

C-3PO What is the name of the gold-plated, humanoid robot in “Star Wars”? (BB-8, K-2SO, R2-D2)

*Chameleon What is the name of the lizard that changes its color to match its surroundings? (Gecko, Iguana, Komodo)

Cherry What is the last name of the former coach commentator who wore wild suits in the “Coach’s Corner” segment of *Hockey Night in Canada*? (Cole, Daniels, MacLean)

China Of which country is Mandarin the primary language? (Hong Kong, Singapore, Taiwan)

CN Tower What is the name of the tallest free-standing structure in Canada? (First Canadian Place, Scotia Plaza, TD Canada Trust Tower)

Crosby What is the last name of the hockey player born in Halifax, Nova Scotia, who serves as the captain of the Pittsburgh Penguins? (Bergeron, Marchand, O’Reilly)

*Curling Which sport uses the terms “stones” and “brooms”? (Cricket, Golf, Rowing)

Da Vinci Who painted the famous portrait of the *Mona Lisa?* (Botticelli, Michelangelo, Raphael)

Edmonton In which Canadian city is located Galaxyland, the largest indoor amusement park in the world? (Calgary, Fort McMurray, Red Deer)

*Einstein What is the last name of the scientist who formulated the theory of relativity? (Bohr, Hawkin, Rutherford)

Elizabeth What is the first name of the current Queen of England? (Anne, Mary, Victoria)

Euro What is the currency used in France? (Franc, Pound, Yen)

*Fossils What is the name of the remains of plants and animals that are found in stone? (DNA, Relics, Skeleton)

Fox What is the name of the Canadian hero who ran across the country with an amputated leg to raise money for cancer research? (Bailey, Drayton, Hansen)

Francis What is the current Pope’s name? (Julius, Leo, Paul)

“Friends” What is the name of the TV sitcom starring Jennifer Aniston and David Schwimmer? (“How I Met Your Mother”, “That ’70s Show”, “The Office”)

Frontenac What is the name of the famous castle overlooking the St. Lawrence River in Quebec City? (Cartier, Champlain, Laurier)

Frosted Flakes™ Which brand of cereal has a tiger as the mascot? (Cheerios™, Sugar Crisp™, Trix™)

Garfield Who is the big, lazy, and orange cartoon cat created by Jim Davis? (Felix, Sylvester, Tom)

Gretzky What is the last name of the Canadian hockey player nicknamed “The Great One”? (Howe, Lemieux, Orr)

Grey Cup What is the Canadian version of the Super Bowl? (Larry O’Brien Trophy, Memorial Cup, Rogers Cup)

Habs (The) What nickname was given to the Montreal Canadiens hockey team? (The B’s, The Dynasty, The Rocket)

*Hibernation What is the name of the long sleep some animals go through during the entire winter? (Dormancy, Huddling, Torpor)

Hitler What was the last name of the Nazi Party leader? (Goering, Himmler, Mengele)

Homer What is the first name of the father in “The Simpsons”? (Barney, Moe, Ned)

*Hook What is the last name of the villainous captain in the fairy tale of “Peter Pan”? (Blackbeard, Flint, Sparrow)

*Houdini What is the name of the famous magician and escape artist who died of appendicitis? (Copperfield, Dante, Mandrake)

Houston What is the last name of the female singer who recorded the song “I Will Always Love You” and was found dead in a bathtub? (Carey, O’Riordan, Winehouse)

Inuksuk What is the name of the man-like silhouette made of stacked stones, that serves as a landmark? (Boundary stone, Effigy, Statuette)

*Javelin What is the name of the spearlike object that is thrown during a track meet? (Discus, Hammer, Shot put)

Ken What is the first name of Barbie’s boyfriend? (Ben, Joe, Ted)

#Kennedy What is the last name of the American president assassinated in 1963? (Lincoln, Nixon, Roosevelt)

King What is the last name of the author who wrote “It” and other horror novels? (Brown, Rice, Rowling)

*Lava/Magma What is the name of the molten rock that runs down the side of a volcano during an eruption? (Clay, Mud, Pumice)

Monroe What is the last name of the famous Hollywood actress and sex symbol of the 1950s, known to have played comedic “blonde bombshell” roles? (Bacall, Hayworth, Mansfield)

Newton What is the last name of the scientist who discovered the law of universal gravitation? (Copernicus, Galileo, Kepler)

Nunavut What is the name of the Canadian territory where the Inuit people live? (Labrador, Northwest Territories, Yukon)

*Odometer What is the name of the car instrument that measures mileage? (Multimeter, Speedometer, Tachometer)

*Ostrich What is the name of the bird that cannot fly and is the largest bird on Earth? (Emu, Penguin, Steamer duck)

Pearson What is the name of the international airport in Toronto? (Bishop, Munro, Trudeau)

Penny What is the first name of the girl next door in “The Big Bang Theory”? (Amy, Bernadette, Emily)

Poppy What do Canadians wear close to their heart to honor veterans prior to, and on, Remembrance Day? (Memorial Ribbon, Royal Pin, Silver Cross)

Poutine What food was created by mixing French fries with cheese curds? (Bangers and Mash, Flipper Pie, Pierogi)

#Presley What is the last name of the American singer known as the “King of Rock ‘n’ Roll”? (Berry, Orbison, Springsteen)

Rideau Canal On which Canadian waterway is found the largest naturally frozen skating rink in the world? (Sault Ste. Marie Canal, Trent Canal, Welland Canal)

Rockies What is the name of the mountain chain in Alberta? (Laurentian, Long Range, St. Elias)

*Ruby What precious gem is red? (Diamond, Emerald, Sapphire)

Saturn What is the name of the planet surrounded by rings? (Jupiter, Mars, Uranus)

Scooby-Doo What is the name of the cartoon dog that helps teenagers solve mysteries? (Goofy, Pluto, Snoopy)

*Shakespeare What is the last name of the author who wrote “Romeo and Juliet”? (Cervantes, Dickens, Molière)

Soviet Union What was the name of Russia during the Cold War? (Leningrad,

/USSR Muscovy, Siberia)

Spielberg What is the last name of the “Jurassic Park” and “Schindler’s List” film director? (Cameron, Scorsese, Tarantino)

Stampede What is the name of the rodeo festival held each year in Calgary? (Frontier Days, Roundup, Western Stock Show)

*Tennis Which sport is associated with the Wimbledon tournament? (F1 auto racing, Soccer, Swimming)

*Titanic What was the name of the supposedly unsinkable ship that sank on its maiden voyage in 1912? (Britannic, Oceanic, Queen Mary)

*Tsunami What is the name of the giant ocean wave caused by an earthquake? (Cyclone, Flood, Hurricane)

Twain What is the last name of the female country singer who grew up in Timmins, Ontario? (Clark, McBryde, Underwood)

Van Gogh What is the last name of the famous painter who allegedly cut off his own ear? (Monet, Picasso, Rembrandt)

#Venice Which Italian city can you visit by sailing on a gondola? (Florence, Naples, Rome)

Voldemort What is the name of Harry Potter’s arch nemesis? (Grindelwald, Malfoy, Snape)

*Wayne What is the last name of Batman’s secret identity? (Kent, Parker, Stark)

Wynne What is the last name of the first woman to become Ontario premier? (Campbell, Cochrane, Notley)
